# Multidimensional 3-Month Follow-Up of Severe COVID-19: Airways beyond the Parenchyma in Symptomatic Patients

**DOI:** 10.3390/jcm11144046

**Published:** 2022-07-13

**Authors:** Matteo Bonato, Piera Peditto, Nicholas Landini, Alessia Fraccaro, Cosimo Catino, Maria Cuzzola, Nicola Malacchini, Francesca Savoia, Nicola Roma, Mauro Salasnich, Martina Turrin, Francesca Zampieri, Giuseppe Zanardi, Fabiola Zeraj, Marcello Rattazzi, Mario Peta, Simonetta Baraldo, Marina Saetta, Michele Fusaro, Giovanni Morana, Micaela Romagnoli

**Affiliations:** 1 Pulmonology Unit, Ca’ Foncello Hospital, Azienda Unità Locale Socio-Sanitaria 2 Marca Trevigiana, 31100 Treviso, Italy; piera.peditto@aulss2.veneto.it (P.P.); alessa.fraccaro@aulss2.veneto.it (A.F.); cosimo.catino@aulss2.veneto.it (C.C.); maria.cuzzola@aulss2.veneto.it (M.C.); nicola.malacchini@aulss2.veneto.it (N.M.); francesca.savoia@aulss2.veneto.it (F.S.); mauro.salasnich@aulss2.veneto.it (M.S.); martina.turrin@aopd.veneto.it (M.T.); francesca.zampieri@aulss2.veneto.it (F.Z.); giuseppe.zanardi@aulss2.veneto.it (G.Z.); fabiola.zeraj@aulss2.veneto.it (F.Z.); micaela.romagnoli@aulss2.veneto.it (M.R.); 2 Department of Cardiac, Thoracic, Vascular Sciences and Public Health, University of Padova, 35122 Padova, Italy; simonetta.baraldo@unipd.it (S.B.); marina.saetta@unipd.it (M.S.); 3 Department of Radiology, Ca’ Foncello Hospital, Azienda Unità Locale Socio-Sanitaria 2 Marca Trevigiana, 31100 Treviso, Italy; nicholas.landini@aulss2.veneto.it (N.L.); nicola.roma@aulss2.veneto.it (N.R.); giovanni.morana@aulss2.veneto.it (G.M.); 4 Department of Radiological, Oncological and Pathological Sciences, Policlinico Umberto I Hospital, “Sapienza” Rome University, 00185 Rome, Italy; 5 Department of Internal Medicine, Ca’ Foncello Hospital, Azienda Unità Locale Socio-Sanitaria 2 Marca Trevigiana, 31100 Treviso, Italy; marcello.rattazzi@aulss2.veneto.it; 6 Department of Emergency, Anesthesiology, Intensive Care, Ca’ Foncello Hospital, Azienda Unità Locale Socio-Sanitaria 2 Marca Trevigiana, 31100 Treviso, Italy; mario.peta@aulss2.veneto.it; 7 Department of Radiology, Oderzo City Hospital, Azienda Unità Locale Socio-Sanitaria 2 Marca Trevigiana, 31046 Oderzo, Italy; fusaro341@gmail.com

**Keywords:** interstitial pneumonia, small airways, fibrosis, bronchiectasis, HRCT

## Abstract

SARS-CoV-2 may lead to a large spectrum of respiratory manifestations, including pulmonary sequelae. We conducted a single-center longitudinal study of survivors from severe COVID-19 cases who underwent a chest CT during hospitalization (CT_H_). Three months after being discharged, these patients were evaluated by a clinical examination, pulmonary function tests and a chest-CT scan (CT_FU_). Sixty-two patients were enrolled. At follow-up, 27% complained of exertional dyspnoea and 12% of cough. Dyspnoeic patients had a lower forced expiratory flow (FEF)_25–75_ (*p* = 0.015), while a CT scan (*p* = 0.016 showed that patients with cough had a higher extent of bronchiectasis. Lung volumes and diffusion of carbon monoxide (DLCO) at follow-up were lower in patients who had been invasively ventilated, which correlated inversely with the length of hospitalization and ground-glass extension at CT_H_. At follow-up, 14.5% of patients had a complete radiological resolution, while 85.5% presented persistence of ground-glass opacities, and 46.7% showed fibrotic-like alterations. Residual ground-glass at CT_FU_ was related to the length of hospitalization (r = 0.48; *p* = 0.0002) and to the need for mechanical ventilation or high flow oxygen (*p* = 0.01) during the acute phase. In conclusion, although patients at three months from discharge showed functional impairment and radiological abnormalities, which correlated with a prolonged hospital stay and need for mechanical ventilation, the persistence of respiratory symptoms was related not to parenchymal but rather to airway sequelae.

## 1. Introduction

Coronavirus disease 2019 (COVID-19) caused by the new coronavirus 2 (SARS-CoV-2), began to spread in China in late-2019, and subsequently all over the world in 2020. SARS-CoV-2 may lead to a large spectrum of respiratory manifestations, from a flu-like syndrome to bilateral pneumonia with acute respiratory failure. Typical CT findings of COVID-19 pneumonia during the acute phase are ground-glass opacities, consolidations, and crazy paving, mainly displaying lower lobe and peripheral distribution [1]. *Wei et al*. [2] described for the first time (May 2020, PubMed) the presence of fibrotic sequelae in CT-scans of 59 patients hospitalized for COVID-19 one month after discharge. Later that year in August, *Zaho et al.* reported functional impairment and persistence of ground-glass opacities, crazy paving, and interstitial thickening in 55 COVID-19 survivors three months after discharge [3]. In 2002, radiological abnormalities (reticular fibrotic-like sequelae) and functional impairment were described in 36% of severe acute respiratory syndrome (SARS) patients [4,5], and in 33% of those with Middle East respiratory syndrome (MERS) also [6]. From the pandemic outbreak, several studies worldwide investigated the functional and radiological sequelae of COVID-19. However, the methodological approaches were dissimilar, and the results were not always consistent; thus, it was difficult to draw exhaustive conclusions. Moreover, only few studies longitudinally examined the evolution of radiological abnormalities by comparing CT scan imaging between the acute phase and follow-up.

From March 2020 to April 2021, the pulmonology intermediate care unit of Ca’ Foncello Hospital (Treviso) in North-east of Italy, treated more than 500 patients affected by severe respiratory failure secondary to COVID-19.

The aims of the study were to assess the presence of respiratory sequelae in a clinical-functional and radiological short-term follow-up of severe COVID-19 survivors —in particular, the evolution of CT abnormalities—, to explore possible predictors of radiological–functional evolution and to investigate potential correlations between clinical presentation and radiological–functional features at follow-up.

## 2. Materials and Methods 

### 2.1. Study Design

We performed a prospective study on a three-month clinical, functional and radiological follow-up of patients admitted to the respiratory intermediate care unit at Ca’ Foncello Hospital (Treviso, Italy) for acute respiratory failure secondary to COVID-19, from March 2020 to April 2021. The study was approved by our referral ethics committee (No. 793 CE/Marca date 9 April 2020). Radiological and functional examinations at baseline and at follow-up both had been performed for a primary clinical purpose accordingly to clinical indications.

Inclusion criteria were (1) a diagnosis of acute respiratory failure (PaO_2_ < 60 mmHg in room air at ABG) secondary to bilateral pneumonia, (2) confirmed SARS-CoV-2 infection by real-time reverse transcription–polymerase chain reaction from a nasopharyngeal swab, (3) appropriate clinical indications for a chest CT scan during hospitalization, and (4) survival to hospitalization.

Exclusion criteria were (1) previous known chronic respiratory comorbidities, (2) low CT scan quality or presence of massive pneumothorax/pleural effusion, (3) death after discharge before follow-up visit, (4) poor clinical conditions after discharge that precluded a visit, (5) refusal to participate in baseline or follow-up examinations, and (6) unreachable after discharge (i.e., tourists).

Past medical history and clinical data at hospital admission were collected by a respiratory physician who analyzed the electronic patients’ registry. Demographics (smoking, comorbidities, laboratory tests, lymphocyte and neutrophil counts, c-reactive protein (CRP), lactate, lactate dehydrogenase, D-dimer and PaO_2_/fraction of inspired oxygen ratio (P/F) at COVID-area arrival), complications, medical treatment, respiratory support, length of hospitalization and length of invasive ventilation were recorded. All patients included in the study received pharmacological treatment and respiratory support based on updated national guidelines at the time of hospitalization [7,8]. Non-invasive and invasive mechanical ventilation parameters were similar for all patients according to national guidelines [8].

We performed a clinical, functional and radiological follow-up after three months from discharge (mean: 110 days). An expert respiratory physician (MB or PP) interviewed patients about general and respiratory symptoms. Exertional dyspnoea was quantified on the mMRC dyspnoea scale. Patients were considered symptomatic if they reported symptoms persisting after discharge and having a negative impact on daily activity or on night rest. At follow-up, patients underwent spirometry, diffusion lung CO and peripheral blood oxygen saturation (SpO_2_) measurement. Pulmonary function tests were performed following ERS/ATS guidelines [9].

### 2.2. CT Acquisitions and Image Analysis

CTs were performed at two centers depending on the phase of the study. During hospitalization (CT_h_), it was the radiology department of the Ca’ Foncello Hospital, which used an OPTIMA 64O (GE Healthcare, Chicago, IL, USA)—tube voltage 120 kV; tube current modulation (range 100–350 mA); slice thickness 1.25 mm; and the reconstruction filter Bone Plus—and a SOMATOM FLASH (Siemens, Ergangen, Germany): tube voltage 120 kV;, tube current modulation (reference 150 mA); slice thickness 1 mm; reconstruction kernels L70F; and very sharp ASA.

Follow up CT (CT_FU_) were performed at the Ca’ Foncello Hospital with the same scanners and parameters or at the Oderzo City Hospital (Oderzo, Italy) on a scan system CT Revolution EVO (GE, Milwaukee, WI, USA); tube voltage 120 kV, tube current modulation (range 100–350 mA range); slice thickness 0.625 mm; and reconstruction filter Type Lung. All high-resolution computed tomography (HRCT) was acquired in full inspiration; no contrast media was administrated.

At the beginning, a general radiologist with 30 years of experience (GM), excluded HRCT images of low quality caused by movement/respiratory artefacts, massive pneumothorax or pleural effusion, which, as a compressive atelectasis, could have affected lung evaluation. Then, a chest radiologist with eight years of experience (NL) visually assessed the extent of lung alterations of both the baseline and follow-up CT and adopted the following semi-quantitative score [10]: each lobe was scored from 0 to 5 based on involvement percentage (0, no involvement; 1, ≤5%; 2, 6–25%; 3, 26–50%, 4, 51–75%; and 5, ≥75%). The total score was the sum of all lobe scores. Both acute lung alterations (ALAs) and fibrotic-like abnormalities (FIBs) were scored in all CTs, and the finding of each one was considered to be part of an ALA based on the most common CT findings described in the literature [1]. In addition, the following were independently assessed: ground glass opacities (GG), crazy paving (CP) pattern and consolidation (CON). The following CT findings were considered as FIBs: parenchymal bands, subpleural lines or reticulation with bronchiectasis (BRN) or architectural distortions, and honeycombing. BRN was separately assessed as being in the severe range of the Brody scoring system [11], customized for cystic fibrosis and based on the extent and severity in each lobe. All alterations were defined according to the glossary of terms for thoracic imaging of the Fleischner Society [12]. All CTs were anonymized and randomized so that, the reader would be unaware of the acquisition date and clinical information. The CTs were evaluated using a standard lung window. One month later 1/3 of the HRCT scans, randomly selected, were reassessed to compute the intrareader agreement, which was scored by a thoracic radiologist with 15 years of experience (NR).

### 2.3. Statistical Analysis

Variables were presented with frequencies and percentages for categorical variables, as median (1st–3rd quartile) or mean ± standard deviation for continuous variables. The difference in explanatory variables was assessed using a Chi-squared or Fisher test for dichotomous and categorical variables, a Student’s *t*-test for normally distributed continuous variables and a Mann–Whitney U or Kruskal–Wallis test for non-normal distributed continuous variables. Correlations between continuous variables was assessed by a Spearman’s test. Intra- and interobserver agreement was evaluated by Cohen’s kappa test: *p* < 0.05 was considered significant. Statistical analyses were performed with SPSS (IBM SPSS Statistics version 23) (IBM, Armonk, NY, USA).

## 3. Results

Of the 589 patients admitted between March 2020 and April 2021 for acute respiratory failure due to COVID-19, a total of 175 (29.7%) met the inclusion criteria. Of these, 113 (64.5%) were excluded for previously reported criteria. Specifically, 82 did not attend the follow-up visit; 16 had previous respiratory comorbidities—COPD (*n* = 5), lung cancer (*n* = 3), bronchiectasis (*n* = 3), pulmonary fibrosis (*n* = 2), severe asthma (*n* = 1), previous lung transplant (*n* = 1), and severe obesity–hypoventilation syndrome (*n* = 1)—and 15 were excluded for radiological criteria. Thus, 62 patients (10.5%) were enrolled.

### 3.1. Clinical-Radiological Characteristics at Hospital Admission and Hospitalization

All demographic and clinical characteristics of the 62 patients at baseline are reported in Table 1. Most were males (45; 72.5%) with a median age of 71 years. The majority of subjects never smoked (58.1%), while only two were current smokers. The most common comorbidity was hypertension (58%). At hospital admission, patients presented a median P/F ratio of 213, median levels of CRP (9.3 (4.3–14.3) mg/L), LDH (369 (312–451) U/L) and D-dimer (813 (476–1288) ng/dL) were higher than normal, while median blood lymphocytes (830 (646–1080) cell/μL) were reduced. Median lactate (1.3 (0.9–1.9) mmol/mol) and blood neutrophil counts (6540 (4310–8852) cell/μL) were in the normal range. Two-thirds of patients needed mechanical ventilation during hospitalization (40.3% non-invasively, and 27.5% invasively), while the remaining 1/3 were on oxygen support only; 17.7% by conventional oxygen support, and 14.5% by high flow nasal cannulas. During hospitalization, 48.3% of patients had no complications; among the residual 51.7%, the most common complication was hyperglycemia (19.3%), followed by superimposed bacterial respiratory tract infection (9.6%), and pulmonary embolism, bacterial sepsis and arrhythmias (8% for all three). Fifty-one patients (83.6%) underwent intravenous steroid treatment: 76.4% with methylprednisolone and 23.6% with dexamethasone. Only 6 patients (9.6%) underwent treatment with tocilizumab, while 24 (38.7%) were given antivirals. The median length of hospitalization was 15 days.

As reported in Table 2, CT_H_ provided the following scores: ALA (13.6 ± 5.5), GG (10.4 ± 4.6), CP (3.7 ± 3.6) and CON (5.9 ± 3.9). FIB (2.7 ± 2.4) and BRN (1.2 ± 2.1. Intra- and inter-reader agreement varied accordingly with the score considered (Cohen’s kappa range 0.71–0.99).

### 3.2. Clinical-Functional-Radiological Characteristics at Follow-Up

**Figure 1:**
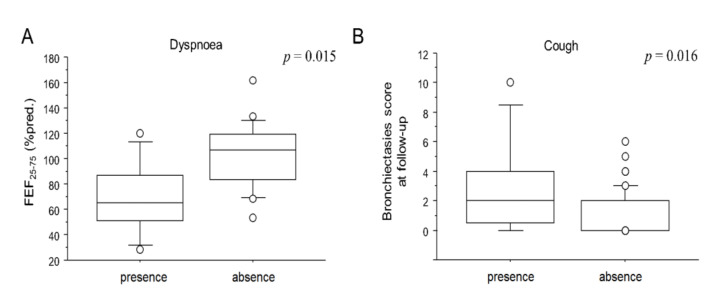
Box plots comparison between the FEF_25-75_ and dyspnoea (**A**) and between BRN and cough (**B**) at follow-up visit. Solid line represents the median; bottom and top of the boxes are the 25th and 75th percentiles; brackets correspond to the 10th and the 90th percentiles.

Clinical and functional characteristics of patients at follow-up are reported in Table 3. Almost half (56.5%) did not report any symptoms. Among symptomatic patients, 17 (27.4%) reported persistent exertional dyspnoea, 8 (12.9%) reported cough and 6 (9.6%) reported fatigue. Symptoms at follow up were not significantly related to CT parenchymal abnormalities (both CT_H_ and CT_FU_), or to lung volumes or DLCO. However, as shown in Figure 1A, patients who reported dyspnoea at follow-up showed a reduced FEF_25–75_ (70.7 ± 30.2 vs. 102.1 ± 26.4% pred.; *p* = 0.015). Moreover, patients who reported cough showed a higher bronchiectasis score at follow-up (2.8 ± 3.3 vs. 1.1 ± 1.5; *p* = 0.016; Figure 1B). No patient had residual chronic respiratory failure. Fifty patients out of 62 (78.1%) underwent pulmonary function tests. Only 11 (24%) reported a functional respiratory disease: 7 patients had a restrictive disease while 4 had an obstructive one. The lung volume means were at the limit of normality. On the other hand, 40% of patients had a mild impairment of alveolar–capillary diffusion (mean 70 ± 14.3%). No associations were observed between radiological and functional impairment at follow-up. As reported in Table 2, CT_FU_ provided the following scores: ALA (7.1 ± 5.7), GG (6.9 ± 5.4), CP (0.9 ± 2.2) and CON (0.3 ± 1.1). FIB (4.2 ± 3.6) and BRN (1.4 ± 2.0) had already been recorded during the acute phase. Intra- and inter-reader agreement varied accordingly with the score (Cohen’s kappa range 0.81–0.99).

### 3.3. Longitudinal Functional-Radiological Evolution from Hospitalization to Follow-Up

#### 3.3.1. Evolution of Acute CT Abnormalities

A complete resolution of all acute abnormalities was observed in only 9 patients (14.5%). In the remaining cases, 53 acute abnormalities (85.5%) were still present at CT_FU._ However, a significant radiological decrease of mean ALA scores was overall observed from CT_H_ and CT_FU_ (−6.4 ± 6.3; *p* < 0.0001). More specifically, a significant reduction was observed for all three acute abnormality scores: GG (−3.5 ± 5.9; *p* = 0.0002), CP (−2.2 ± 3.5; *p* < 0.0001) and CON (−5.6 ± 3.9; *p* < 0.0001). Of note, while CP and CON were still present in only a minority of patients (*n* = 15 and *n* = 7, respectively), with a wide extent reduction in percentage between CT_H_ and CT_FU_ (−74 ± 74% and −94 ± 32%, respectively), GG was still present in 85.5% of patients (*n* = 53), and its reduction in percentage was lower in comparison to the other two scores (−31 ± 56%).

**Figure 2:**
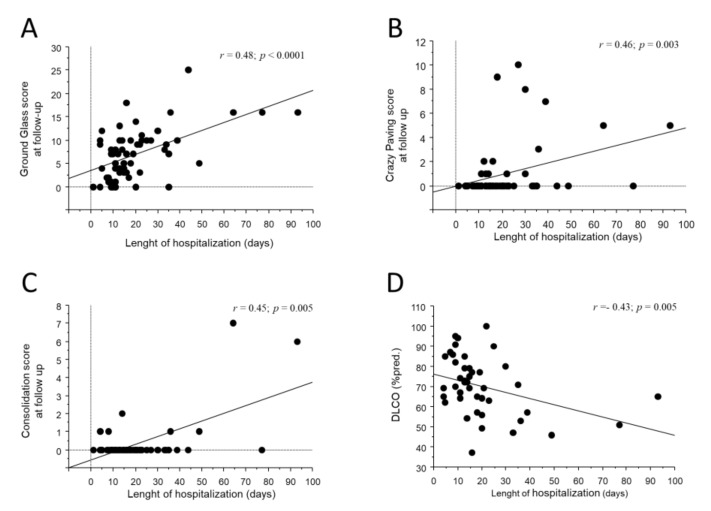
Bivariate scatterplots reporting the relationship between the length of hospitalization (***x*** axes) and GG (**A**), CP (**B**), CON (**C**) at CT_FU_ and DLCO (**D**) at follow-up visit. Regression line is represented.

**Figure 3:**
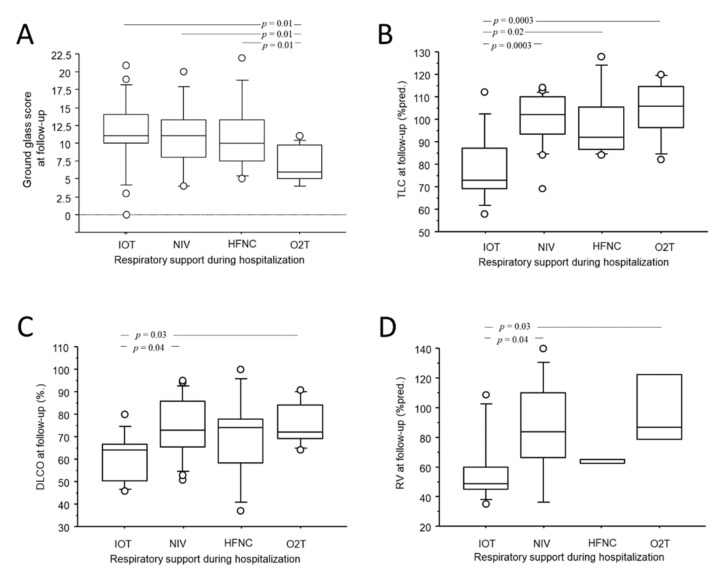
Box-plot comparison of GG at CT_FU_ (**A**), the TLC (**B**), the DLCO (**C**), the RV (**D**) at follow-up with respiratory support needed during hospitalization. Solid line represents the median; bottom and top of the boxes are the 25th and 75th percentiles; brackets correspond to the 10th and the 90th percentiles.

As shown in Figure 2A–C, the length of hospitalization moderately correlated with all three acute abnormalities (r = 0.48; *p* < 0.0001 for GG; r = 0.46; *p* = 0.0003 for CP; r = 0.45; *p* = 0.005 for CON) as well as to the residual ALA score at CT_FU_ (r = 0.48; *p* = 0.0002). Residual GG at CT_FU_, but not CON or CP, was significantly lower in patients who required only conventional O_2_T support during hospitalization (2.9 ± 3.7 for conventional O_2_T vs. 8.8 ± 6.7 for IV, 7.0 ± 4.4 for NIV and 8.0 ± 5.1 for HFNC vs.; *p* = 0.01 Figure 3A). No associations were observed between the persistence of acute abnormalities at follow-up with gender, age, comorbidities, laboratory tests, complications, length of invasive ventilation, and pharmacological treatment during hospitalization.

#### 3.3.2. Development of Chronic CT Abnormalities

Fibrotic-like alterations and bronchiectasis were already observed during hospitalization. At follow-up, fibrotic-like alterations were observed in 29 patients (46.7%), although the extent was modest in comparison to the residual acute alterations (CT_FU_: FIB 4.2 ± 3.6 vs. TAP 7.1 ± 5.7; *p* = 0.0002). Despite this, a significant increase in FIB was observed from CT_H_ to CT_FU_ (+1.6 ± 3.3; *p* = 0.001). Conversely, BRN did not significantly increase from CT_H_ to CT_FU_. FIB at CT_FU_ was weakly but significantly correlated to the ALA score at CT_H_ (r = 0.25; *p* = 0.04). No other acute CT abnormalities, nor gender, age, comorbidities, complications, pharmacological treatment, respiratory support, length of invasive ventilation, and laboratory tests during hospitalization, were predictive of FIB at CT_FU_.

#### 3.3.3. Development of Functional Impairment

GG at CT_H_ correlated inversely with total lung capacity, residual volume and DLCO at follow-up (r = −0.45; *p* = 0.003 for TLC; r = −0.42; *p* = 0.01 for RV; r = −0.48; *p* = 0.02 for DLCO). The length of hospitalization also correlated inversely with DLCO at follow-up (r = −0.43; *p* = 0.0005, Figure 2D).

As shown in Figure 3B–D, patients who had been invasively ventilated during hospitalization showed significantly reduced total lung capacity (78 ± 15.6%pred. for IV vs. 97 ± 15.6%pred. for HFNC and 99 ± 12%pred. for NIV and 89 ± 13.6%pred. for conventional O_2_T; *p* = 0.005), residual volume (58 ± 24.4%pred. for IV vs. 64 ± 1.7%pred. for HFNC and 86 ± 33%pred. for NIV and 99 ± 30%pred. for conventional O_2_T; *p* = 0.005) and DLCO (60 ± 10.8%pred. for IV vs. 69 ± 19.7%pred. for HFNC and 74 ± 13.1%pred. for NIV and 76 ± 10%pred. for conventional O_2_T; *p* = 0.03) at follow up. No associations were observed between functional impairment at follow-up and gender, age, comorbidities, complications, pharmacological treatment, length of invasive ventilation, and laboratory tests during hospitalization.

## 4. Discussion

The present study analyzed the clinical, functional and radiological evolution of severe COVID-19 three months from discharge in 62 patients. We demonstrated for the first time that the persistence of symptoms was not related to a residual parenchymal disease, but rather to an increased bronchiectasis extension and to a FEF_25-75_ impairment, as manifestation of airways abnormalities. At follow-up, the functional impairment and residual, acute radiologic alterations were related to the severity of acute COVID-19 pneumonia in terms of length of hospitalization and intensive respiratory support. Radiological acute abnormalities, especially ground-glass opacities, were still present in the majority of patients (85%) after the three months. Finally, the increase in fibrotic-like alterations was modest although present in 50% of patients.

About half of the patients complained of respiratory symptoms at follow-up, and unlike what was assumed, we observed a discrepancy between respiratory symptom with lung volumes/alveolar-capillary diffusion and residual radiological parenchymal impairment. These findings were consistent with previous studies that observed only a weak correlation between post-COVID respiratory symptoms and functional-radiological parenchymal impairment at follow-up [13,14,15]. Indeed, there is still a lack of evidence regarding the mechanisms of post-COVID respiratory syndrome.

In our cohort, dyspnoeic patients showed impaired FEF_25–75_, which was a sign of small-airways disease, whereas patients who reported cough showed increased bronchiectasies extension in the CT scan. The first result was consistent with the findings of other authors, who demonstrated on a paired inspiratory/expiratory CT scan that patients with persistent respiratory symptoms after acute COVID-19 showed air trapping [16,17]. Bronchiectasis in COVID-19 patients had already been described both during hospitalization and at follow up and seemed to be associated with a more severe infection [14,18]. Indeed, all these results suggested that the respiratory symptoms in patients with “long COVID-19” might be due to airway abnormalities rather than, or not exclusively to, lung parenchymal sequelae. These data deserve to be further investigated in larger cohorts since they may play an important role in patient management during follow-up.

Several studies have analysed CT parenchymal imaging of patients hospitalized for COVID-19 in a short- and medium-term follow-ups [2,3,13,14,15,19,20,21,22]. The methodological differences of these previous studies make a direct comparison among data difficult. However, the persistence of residual radiological lung abnormalities after discharge was reported by all authors with wide variable prevalence, ranging from 20 [15] to 97.7% [19]. Our results (85.5%) ranked among the higher values probably because a more severe disease occurred in our cohort: indeed, 2/3 of patients required mechanical ventilation support during hospitalization. Thus, we observed a moderate correlation between the severity of COVID-19 during the acute phase -in terms of length of hospitalization or need for mechanical ventilation- and the parenchymal sequelae at follow-up -both functional and radiological- confirming what was previously reported by other authors [13,14,15]. Similar to other papers, ground-glass persistence at follow-up was the most frequently observed CT abnormality [2,3,13,14,15,19,20,21,22], while consolidations recovered almost completely [14,15,19,21]. Concerning fibrotic-like alterations, our results were also in accordance with previous reports, which showed it being present in fewer than 50% of patients [2,14,15,19]. However, unlike the others, we did not observe any correlation at follow-up between the severity of acute phase and fibrotic-like extension [13,15]. In our population, while acute abnormalities showed a decreasing trend from acute phase to follow-up, there was a significant, although modest, increase of fibrotic-like alterations. Whether these findings represent early signs of pulmonary fibrosis is under debate [23,24,25]. Concerning our results, fibrotic-like alterations were also observed in the acute phase; therefore, it was difficult to determine whether these patients actually developed pulmonary fibrosis. We speculated that these lesions were likely not present before developing COVID-19 as the majority of our patients were less than 70 years old, non-smokers, and without previous chronic respiratory disease.

Our study had some limitations. First of all, the results were obtained for a relatively small number of patients and need to be confirmed by a larger cohort. Second, the study was conducted while facing the pandemic, and the rapid evolution of national guidelines on COVID-19 treatment did not allow the same protocols of pharmacological treatment for the whole cohort. Therefore, it was impossible to assess the possible influence of drug treatment in the acute phase on follow-up residual impairment/recovery. Finally, we did not use questionnaires for interviewing patients’ symptoms at follow-up because symptom assessment was not the focus of our study; however, we are confident that considering only symptoms that were persistent and had a tangible impact on patients’ lives provided an accurate assessment.

In conclusion, we performed a multidimensional assessment of severe COVID-19 survivors three months form hospital discharge. We reported for the first time that the persistence of respiratory symptoms were related to airway sequelae rather than to parenchymal alterations. This finding might deserve to be investigated during a longer follow-up, to evaluate whether it might be a target for a pharmacological approach.

Lung parenchymal abnormalities persisted at short follow-ups both on the functional and radiological side and were related to the severity of the acute disease for length of hospitalization and need for mechanical ventilation. Finally, the clinical and pathological meaning of fibrotic-like alterations remains under debate, and further investigations are needed to clarify their role.

## Figures and Tables

**Table 1:** Demographics, anamnestic data, laboratory tests, respiratory support and pharmacological treatment during hospitalization.

Subjects, *n*	62
Gender (male/female), *n* (%)	45 (72.5)/17 (27.5)
Age	68.5 ± 9.5
Smoke habit, current/former/never, *n* (%)	2 (3.2)/24 (38.7)/36 (58.1)
Comorbidities, *n* (%)	
Hypertension	36 (58)
Dyslipidemia	19 (30.6)
Diabetes	17 (27.4)
Obesity	11 (17.7)
Cardiopathy	12 (19.3)
Active malignancy	2 (3.2)
Chronic kidney disease	2 (3.2)
P/F, ratio	213 (143–285)
CRP, mg/L	9.3 (4.3–14.3) (nv < 1)
Lactate, mmol/L	1.3 (0.9–1.9) (nv < 2.3)
LDH, U/L	369 (312–451) (nv < 280)
D-dimer, ng/mL	813 (476–1288) (nv < 300)
Blood lymphocytes, cell/mcl	830 (646–1080) (nv 1000–4800)
Blood neutrophyls, cell/mcl	6540 (4310–8852) (nv 1500–8000)
Respiratory support, *n* (%)	
Conventional O_2_T	11 (17.7)
HFNC	9 (14.5)
NIV	25 (40.3)
IV	17 (27.5)
Complications during hospitaization, *n* (%)	
Pulmonary embolism	5 (8.0)
Deep venin thrombosis w/o PE	2 (3.2)
Superimposed bacterial respiratory tract infection	6 (9.6)
Superiomposed bacterial urinary tract infection	3 (4.8)
Superimposed bacterial biliary tract infection	2 (3.2)
Bacterial sepsis	5 (8.0)
Fungal sepsis	1 (1.6)
Superimposed CMV infection	4 (6.4)
Hyperglicemia	12 (19.3)
Acute hearth failure	4 (6.4)
Acute kidney failure	2 (3.2)
Acute liver failure	0 (0%)
Arrhythmias	5 (8.0)
Stroke	2 (3.2)
None comomplication	30 (48.3)
Steroid treatment, *n* (%)	51 (83.6)
Methylprednisone	39 (76.4)
Dexamethasone	12 (23.6)
Tocilizumab treatment, *n* (%)	6 (9.6)
Antiviral treatment, *n* (%)	24 (38.7)
Remdesevir	18 (75)
Lopinavir/ritonavir	6 (25)
Length of hospitalization, days	15 (11–23)
Length of invasive ventilation, days	8 (4.7–10)

Data are expressed as *n* (%) or mean ± SD or median (IQR). nv: normal value. P/F: PaO2/FiO2 ratio; CRP: C-Reactive Protein; LDH: lactate dehydrogenases; HFNC: high flow nasal cannulas; NIV: non-invasive ventilation; IV: invasive ventilation; PE: pulmonary embolism; CMV: cytomegalovirus.

**Table 2:** CT scan abnormalities during hospitalization (CT_H_) and at follow-up (CT_FU_).

	CT_H_	CT_FU_	Δ_FU-H_	*p*-Value
Total acute lung alterations (ALA)	13.6 ± 5.5	7.1 ± 5.7	−6.4 ± 6.3	<0.0001
Ground glass (GG)	10.4 ± 4.6	6.9 ± 5.4	−3.5 ± 5.9	0.0002
Crazy paving (CP)	3.7 ± 3.6	0.9 ± 2.2	−2.2 ± 3.5	<0.0001
Consolidation (CON)	5.9 ± 3.9	0.3 ± 1.1	−5.6 ± 3.9	<0.0001
Fibrotic-like alterations (FIB)	2.7 ± 2.4	4.2 ± 3.6	+1.6 ± 3.3	0.0003
Bronchiectasis (BRN)	1.2 ± 2.1	1.4 ± 2.0	+0.2 ± 1.8	n.s.

Data are expressed as mean ± SD, *p*-value resulted from ***t***-test. Δ_FU-H_ is the difference between the score at follow-up and during hospitalization; n.s. non-significant.

**Table 3:** Clinical findings and respiratory function at follow-up visit.

No Symptoms, *n* (%)	35 (56.4)
Exertional dyspnoea	17 (27.4)
Cough	8 (12.9)
Fatigue	6 (9.6)
Sputum	2 (3.2)
Others (ageusia, chest pain, parenthesis, muscle and joint pain)	0
mMRC	0 (0−1)
Respiratory failure, *n* (%)	0 (0)
Pulmonary function test performed, *n* (%)	50 (78.1)
Obstructive disease, *n* (%)	4 (8)
Restrictive disease, *n* (%)	7 (14)
Mixed disease, *n* (%)	0 (0)
FEV1/FVC, %pred.	79 ± 6.9 (nv > 70)
FEV1 %pred.	109 ± 15.6 (nv > 80)
FVC %pred.	109 ± 16.6 (nv > 80)
FEF 25–75%pred.	94 ± 28.7 (nv > 80)
TLC %pred.	94 ± 16.3 (nv 80–120)
RV %pred.	75 ± 30.8 (nv > 80)
DLCO %pred.	70 ± 14.3 (nv > 80)

Data are expressed as *n* (%) or mean ± SD or median (IQR); nv: normal value. FEV1: Forced expired volume in the first second of expiration; FVC: Forced vital capacity; FEF: Forced expired flow at 25–75% of forced vital capacity; TLC: Total lung capacity; RV residual volume; DLCO: diffusion of carbon monoxide.

## Data Availability

The data that support the findings of this study are available from the corresponding author, M.B., upon reasonable request.

## Abbreviations

ABG	Arterial blood gas analysis
ARDS	Acute respiratory distress syndrome
CON	Consolidation
CP	Crazy paving
CRP	C-reactive protein
DLCO	Diffusion of carbon monoxide
FIB	Fibrotic-like alterations
GG	Ground glass
HFNC	Heated humidified high-flow oxygen therapy
CT	Chest computed tomography
ICU	Intensive care unit
IQR	Interquartile range
IV	Invasive ventilation
FEV1	Forced expired volume in the first second of expiration
FiO_2_	Fraction of inspired oxygen
FVC	Forced vital capacity
K–W	Kruskal–Wallis test
LDH	Lactate dehydrogenase
mMRC	Modified medical research council
PaO_2_	Arterial partial pressure of oxygen
P/F	PaO_2_/FiO_2_ ratio
NIV	Non-invasive ventilation
NV	Normal values
O_2_T	Oxygen therapy
RV	Residual volume
SD	Standard deviation
ALA	Acute lung alterations
SARS-CoV-2	Severe acute respiratory syndrome coronavirus 2
TLC	Total lung capacity
